# Individual biological sensitivity to environmental influences: testing the differential susceptibility properties of the 5HTTLPR polymorphism in relation to depressive symptoms and delinquency in two adolescent general samples

**DOI:** 10.1007/s00702-018-1854-8

**Published:** 2018-02-09

**Authors:** Cecilia Åslund, Kent W. Nilsson

**Affiliations:** Centre for Clinical Research Västerås, Västmanland County Hospital Västerås, Uppsala University, 721 89 Västerås, Sweden

**Keywords:** Antisocial behaviour, Depression, Emotion regulation, Gene–environment interaction, Human, SERT, SLC6A4

## Abstract

The gene–environment interaction research field in psychiatry has traditionally been dominated by the diathesis–stress framework, where certain genotypes are assumed to confer increased risk for adverse outcomes in a stressful environment. In later years, theories of differential susceptibility, or biological sensitivity, suggest that candidate genes that interact with environmental events do not exclusively confer a risk for behavioural or psychiatric disorders but rather seem to alter the sensitivity to both positive and negative environmental influences. The present study investigates the susceptibility properties of the serotonin transporter-linked polymorphic region (5HTTLPR) in relation to depressive symptoms and delinquency in two separate adolescent community samples: *n* = 1457, collected in 2006; and *n* = 191, collected in 2001. Two-, three-, and four-way interactions between the 5HTTLPR, positive and negative family environment, and sex were found in relation to both depressive symptoms and delinquency. However, the susceptibility properties of the 5HTTLPR were distinctly less pronounced in relation to depressive symptoms. If the assumption that the 5HTTLPR induces differential susceptibility to both positive and negative environmental influences is correct, the previous failures to measure and control for positive environmental factors might be a possible explanation for former inconsistent findings within the research field.

## Introduction

The capacity to regulate our emotions and behaviour is important for social functioning. Limitations in emotion and behaviour regulation could be both genetically and environmentally transmitted. Here, we test a sex-diverse variant of an evolutionary approach (based on maximal reproductive genetic variability) of the differential susceptibility hypothesis, postulating that for the serotonin transporter-linked polymorphic region (5HTTLPR), the putative ‘plasticity alleles’ differ between adolescent boys and girls, and that the more positive and negative environmental influences an individual experiences, the more the emotion-regulated phenotypic expression will differ.

### The function of serotonin in emotion-regulation processes

Serotonin (5-hydroxytryptamine, 5-HT) is one of the most widely distributed neurotransmitters in the brain. It is present in all bilateral animals. In humans, it influences a variety of behavioural and neuroendocrine functions including the sleep–wake cycle, appetite, aggression, sexual behaviour, pain sensitivity, sensorimotor reactivity, and learning (Lucki 1998; Naughton et al. 2000). Serotonin has been suggested as a neurotransmitter of major importance for predicting a wide variety of psychological conditions and behaviours, including aggression, alcoholism, anxiety, novelty seeking, depression, and antisocialism (Shattuck et al. 2014). Experimentally induced alterations in 5-HT pathways cause changes in emotion-related behaviours in animal models (Griebel 1995), and in social cognition (Canli and Lesch 2007) and mood alteration in humans (Walderhaug et al. 2007). As serotonin is a key modulator of emotional behaviour and emotion regulation, any genetic variation that modifies the degree of serotonin signalling in the brain might be expected to contribute to variation in emotional behaviour (Hariri and Holmes 2006; Shattuck et al. 2014). One main regulator of serotonin is the serotonin transporter (5-HTT), which removes the serotonin released into the synaptic cleft. The 5-HTT protein is encoded by the *SLC6A4* gene. Transcriptional activity of the *SLC6A4* gene is modulated by several variations, including the serotonin transporter-linked polymorphic region (5HTTLPR), which is located in the upstream regulatory promoter region of the *SLC6A4*, and consists of different lengths of a repetitive sequence containing 20- to 23-bp-long repeat elements (Canli and Lesch 2007; Lesch et al. 1996; Heils et al. 1996). Insertion or deletion of nucleotide sequences has, at some point in evolution, resulted in a short (S) 14-repeat and a long (L) 16-repeat allele. The short allele has been associated with lower transcriptional efficiency (Canli and Lesch 2007; Lesch et al. 1996; Heils et al. 1996), and in humans, has been associated with anxiety-related personality traits and amygdala hyper-reactivity in emotion-related tasks (Canli and Lesch 2007; Hariri and Holmes 2006; Costafreda et al. 2013). An analogous polymorphism of the 5HTTLPR exists in rhesus macaques (Lesch et al. 1997), which, next to humans, are the most widely distributed primate species. The fact that multiple polymorphisms affecting serotonergic function have evolved and are maintained in both humans and macaques suggests that there is positive evolutionary selection acting on *SLC6A4* and its regulatory regions—these polymorphisms involve some kind of evolutionary advantage (Shattuck et al. 2014).

### Gene–environment interaction and the 5HTTLPR polymorphism

In 2003, Caspi and colleagues presented the ground-breaking finding that stressful life events increased the risk of depression among carriers of the short alleles of the 5HTTLPR (Caspi et al. 2003). Since then, a steadily increasing number of candidate gene (cG) studies have identified significant gene × environment (cG × E) interactions for psychiatric outcomes. However, during recent years, there have been controversies and criticism within the cG×E research field in psychiatry, including failures to replicate findings. Two meta-analyses of 5 (Munafo et al. 2009) and 14 (Risch et al. 2009) studies, respectively, failed to find an interaction between the 5HTTLPR polymorphism and stressful life events for depression. On the other hand, in 2014, a meta-analysis identified 81 studies, and a significant relationship between the S allele of the 5HTTLPR, stress, and depression was confirmed (*p* = 0.0000009) (Sharpley et al. 2014). In the most recent meta-analytic update, Bleys et al showed a small but significant effect of 5HTTLPR in interaction with stress in the prediction of depression [OR (95% CI) = 1.18 (1.09; 1.28)], using data from 51,449 participants (Bleys et al. 2018). However, in a collaborative meta-analysis using original data from each participating study, the conclusion was; “if an interaction exists in which the S allele of 5-HTTLPR increases risk of depression only in stressed individuals, then it is not broadly generalisable, but must be of modest effect size and only observable in limited situations” (Culverhouse et al. 2017). A review of 103 studies of different cG × E interactions published between 2000 and 2009 suggested that the failure to replicate evidence of cG × E associations could be explained by the differences in the design of the studies, the statistical methodologies used, the measurement of outcome variables, the environmental factors that were included, and the presence of publication bias among replication attempts (Duncan and Keller 2011). We and others recently added the dose and direction of the environmental factor being examined, and gene × gene interactions (G × Gs), to this list of considerations (Comasco et al. 2013; Beaver and Belsky 2012; Boyce and Ellis 2005; Belsky and Pluess 2009).

### Different theoretical frameworks in cG × E research

The cG × E research field has traditionally been dominated by the diathesis–stress framework, where certain genotypes are assumed to confer increased risk for adverse outcomes in a stressful environment (Dick 2011; Manuck and McCaffery 2014). In contrast to the diathesis–stress framework, the differential susceptibility hypothesis (Belsky and Pluess 2009) [also referred to as biological sensitivity to context (Boyce and Ellis 2005) or genetic plasticity (Belsky et al. 2009)] suggests that cGs that interact with environmental events do not exclusively confer a risk for behavioural or psychiatric disorders, but rather seem to alter the sensitivity to the environment per se (Belsky and Beaver 2011; Belsky et al. 2009; Belsky and Pluess 2009; Hankin et al. 2011), regarding both positive and negative influences. Carriers of such plasticity alleles who are raised in positive environments show better-than-average positive outcomes, whereas carriers of the same genotypes raised in adverse conditions show negative outcomes, compared with non-carriers (Reiss et al. 2013; Hankin et al. 2011; Nilsson et al. 2014). Such susceptibility effects have been shown in several recent cG × E studies of different cGs when both positive and negative environmental influences have been taken into account. For instance, the association between maltreatment and the 5HTTLPR-SS in relation to depression was significantly reduced when adjusting for social support, such that maltreated children with positive social support had only minimal increases in their depression scores (Kaufman et al. 2004). A large meta-analysis of cG × E effects for the 5HTTLPR and developmental outcomes among children and adolescents up to age 18 showed that carriers of the short allele were more vulnerable to adverse environment exposures but also profited more from positive environments (van IJzendoorn et al. 2012). Among individuals homozygous for the short 5HTTLPR allele, exposure to negative parenting was associated with low positive affect, while exposure to positive parenting was associated with high positive affect (Hankin et al. 2011). Another study showed that 5HTTLPR interacted with both high and low socio-economic statuses in relation to delinquency (Åslund et al. 2013). Furthermore, epistatic as well as epigenetic mechanisms have been suggested to influence 5HTTLPR variation in expression (Iurescia et al. 2017). A three-way interaction between brain-derived neurotrophic factor (*BDNF*) genotype, 5HTTLPR, and maltreatment history was related to depression in children, and this relation was moderated by social support (Kaufman et al. 2006). Another study showed distinct susceptibility effects of the 5HTTLPR, *MAOA*, and *BDNF* genotypes, both in interaction with each other and with positive and adverse environments in relation to adolescent delinquency (Nilsson et al. 2014). Moreover, carriers of the susceptibility alleles who had experiences of maltreatment also had a protective effect from a positive parent–child relationship in relation to delinquent behaviour (Nilsson et al. 2014). A possible evolutionary advantage of the S allele has been suggested in a study showing improved emotional processing in memory and attention in S allele carriers of the 5HTTLPR during tryptophan depletion (Roiser et al. 2007).

**Fig. 1:**
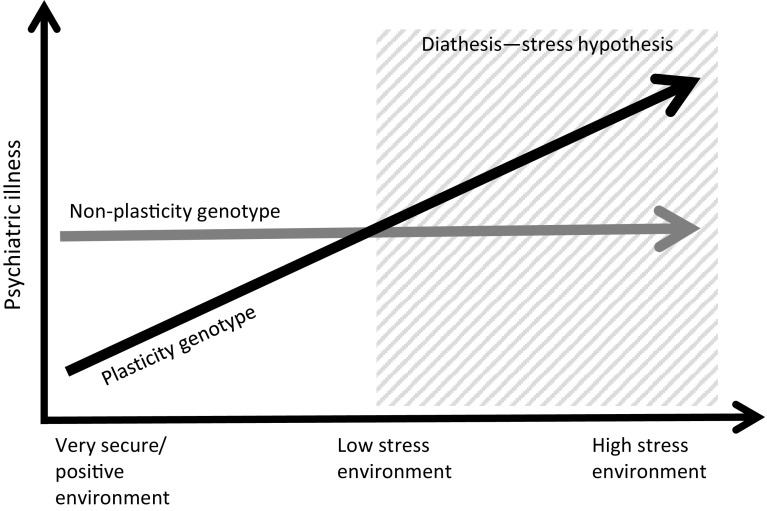
Comparison of the differential susceptibility hypothesis and the diathesis–stress hypothesis

If the differential susceptibility theories of cG × E are correct, the outcomes of previous cG × E studies using the traditional diathesis–stress perspective would be expected to vary depending on the properties of the environmental measurements as well as the psychosocial properties of the study population, as shown in Fig. 1. Studies using the diathesis–stress perspective have traditionally focused on stressful versus non-stressful environments, thereby failing to measure positive environmental factors and consequently limiting the results to interpretations of these genes as “vulnerability genes”, as shown in the right side of Fig. 1. The failure to measure and analyse the effects of genuinely positive environmental factors (which are not equal to the absence of adverse factors) means that the possibly positive effects of the susceptibility genes in supporting and positive environments have been neglected. One implication is that case–control studies will suffer from the confounding effects of different environmental loads in the case versus control samples. Moreover, differential susceptibility effects would mean that meta-analyses of cG × E effects would be at risk of null findings, depending on the protective effects of the positive psychosocial factors that are implicitly included, but not measured, in the “non-stressful” environment of the study populations. It is, therefore, of utmost importance that future studies in the cG × E research field in psychiatry strive to define environmental influences by dynamic measurements that include both positive and negative environmental influences.

### Sex differences in gene–environment interactions and serotonergic function

Findings in the cG × E research field of psychiatry are further complicated by reports of contradictory effects in males and females; the allelic variation associated with increased risk in males has been associated with decreased risk or null findings in females and vice versa. Such sex differences in gene–environment interactions have previously been reported for the 5HTTLPR (Sjoberg et al. 2006; Åslund et al. 2009, 2013; Brummett et al. 2008a). In three recent meta-analyses (Sharpley et al. 2014; Bleys et al. 2018; Culverhouse et al. 2017), efforts have been made to clarify if the S allele of the 5HTTLPR is more frequently associated with depression among females. However, in these analyses, sex has been used as a main effect term, not as an interaction term, thereby not taking different directions of effect into account (Keller 2014). Sex differences have also been suggested for genotype encoding of monoamine oxidase A (MAOA) (Nilsson et al. 2006; Sjoberg et al. 2007; Åslund et al. 2011; Prom-Wormley et al. 2009), which is a key enzyme in the catabolism of serotonin. Moreover, it has been suggested that the serotonin system behaves differently in males and females (Biver et al. 1996; Costes et al. 2005; Williams et al. 2003; Brummet et al. 2008; Walderhaug et al. 2007). For example, the S allele of the 5HTTLPR is associated with higher cerebrospinal fluid levels of the major serotonin metabolite 5-HIAA in women and lower levels in men (Williams et al. 2003). Men with the LL alleles and women with the SS alleles of 5HTTLPR show increases in negative affect during tryptophan infusion (Brummett et al. 2008b), and recent findings show reversed patterns of neural activity in aggression-related brain systems in males and females for the high and low expression alleles of a polymorphism of the *MAOA* gene (Holz et al. 2014). Based upon the incongruent findings from individual studies and different conclusions from meta-analyses regarding the interaction between 5HTTLPR and environmental factors in relation to depression, we suggest that sex might be an important factor for further investigation, and that interaction effects of sex should be evaluated in relation to both environmental and genetic factors.

### Aim

The aim of the present study was to investigate the differential susceptibility hypothesis in relation to the 5HTTLPR and associations with depressive symptoms and delinquency in adolescence. Specifically, the study investigated: (a) biological susceptibility effects of the 5HTTLPR and positive and negative psychosocial factors in relation to (1) depressive symptoms and (2) delinquency; and (b) sex differences and sex interaction effects in relation to the differential susceptibility effects of the 5HTTLPR.

The present study uses two separate general adolescent samples: one from a questionnaire study and one from an interview study. Data from the questionnaire study have been previously published regarding cG × E effects of 5HTTLPR in relation to both depression (Åslund et al. 2009) and delinquency (Åslund et al. 2013). However, the previous publications used the traditional diathesis–stress perspective, failing to investigate the influence of positive environment and presence of differential susceptibility effects. Moreover, the previous studies did not investigate gene × sex interactions, which we now suspect are of importance, given the suggested sex differences in cG × E effects. Thus, the present study further advances the research field by investigating parameters of positive environmental influences according to the differential susceptibility hypothesis, and gene × sex interactions in two separate community samples to investigate differential susceptibility effects. If the differential susceptibility theories of cG × E are correct, they may explain the inconsistent findings within the diatheses–stress-dominated research field of cG × E in psychiatry.

## Materials and methods

### Sample 1: SALVe 2006

#### Participants and procedure

The present study was part of the Survey of Adolescent Life in Västmanland (SALVe), undertaken in 2006. Västmanland is a medium-sized county in Sweden. All students in the second year of high school (age 17–18 years) were asked to complete a questionnaire during class hours, or, if absent, on the subsequent day, and to provide a saliva sample for DNA extraction. Participation was anonymous and voluntary. A total of 2263 students, 77.4% of the target population, completed the questionnaires, of whom 183 were late responders who returned their questionnaires by mail and 2131 provided saliva samples (Åslund et al. 2009). Due to problems with the 5HTTLPR analyses 586 participants were excluded, along with another 88 who had missing answers on the key variables, thereby leaving 1457 participants.

#### Measures

*Sex* Boy (1) or Girl (2).

*Ethnicity* Scandinavian ethnicity was classified as both parents born in Sweden or Scandinavia (1). Non-Scandinavian ethnicity was used when at least one parent was born outside of Scandinavia (2).

*Family subjective socio-economic status* The participants were asked to rate the wealth/income of their family in comparison with the rest of society by the following question: “Imagine society as being like a ladder. At the bottom are those with the least money, at the top are those with the most. If you think about how wealthy your own family is compared with the rest of society, where would you place your family on this scale?”. Answers were given on a 7-point Likert scale ranging from “Least money” (1) to “Most money” (7). The measure is a modified version of the Goodman et al.’s (2001) MacArthur Scale of Subjective Social Status, which has been used and reported previously (Åslund et al. 2013).

*Positive family relations* The participants were asked to respond to the following statements regarding their relationship with their parents: (1) I can talk with my parents about almost everything; (2) I like to be with my parents; (3) I can always trust my parents when it really matters; and (4) my parents give me many opportunities to do fun things with them. Answer alternatives were: totally disagree (0); somewhat disagree (1); neither disagree nor agree (2); somewhat agree (3); and totally agree (4). A positive family relations’ index was created as a summation of the above questions, with a possible score range of 0–16 (Nilsson et al. 2014). The internal consistency of the items measured by Cronbach’s *α* was 0.825.

*Family adversity* Six questions assessed the adolescent’s perception of his/her family: 1. Have you ever run away from home? No (0), Yes (1); 2. Does anyone in your family have a problem with alcohol or narcotics? No (0), Yes (1); 3. Have there ever been any severe, heart-rending quarrels between your parents? Never, or less than once a month (0), Yes, at least once a month (1); 4. Has either of your parents ever pushed, beaten, or used any other kind of violence against the other? Never or seldom (0), at least once a year (1); 5. Has either of your parents ever pushed or beaten you, or used any other kind of violence against you? Never or seldom (0), at least once a year (1); and 6. Have you ever been treated badly psychologically (for example, taunted or scorned) by either of your parents? Never or seldom (0), at least once a year (1). A family adversity index variable was created by a summation of these questions with a possible score range of 0–6 (Nilsson et al. 2011). The internal consistency of the items measured by Cronbach’s *α* was 0.571.

**Fig. 2:**
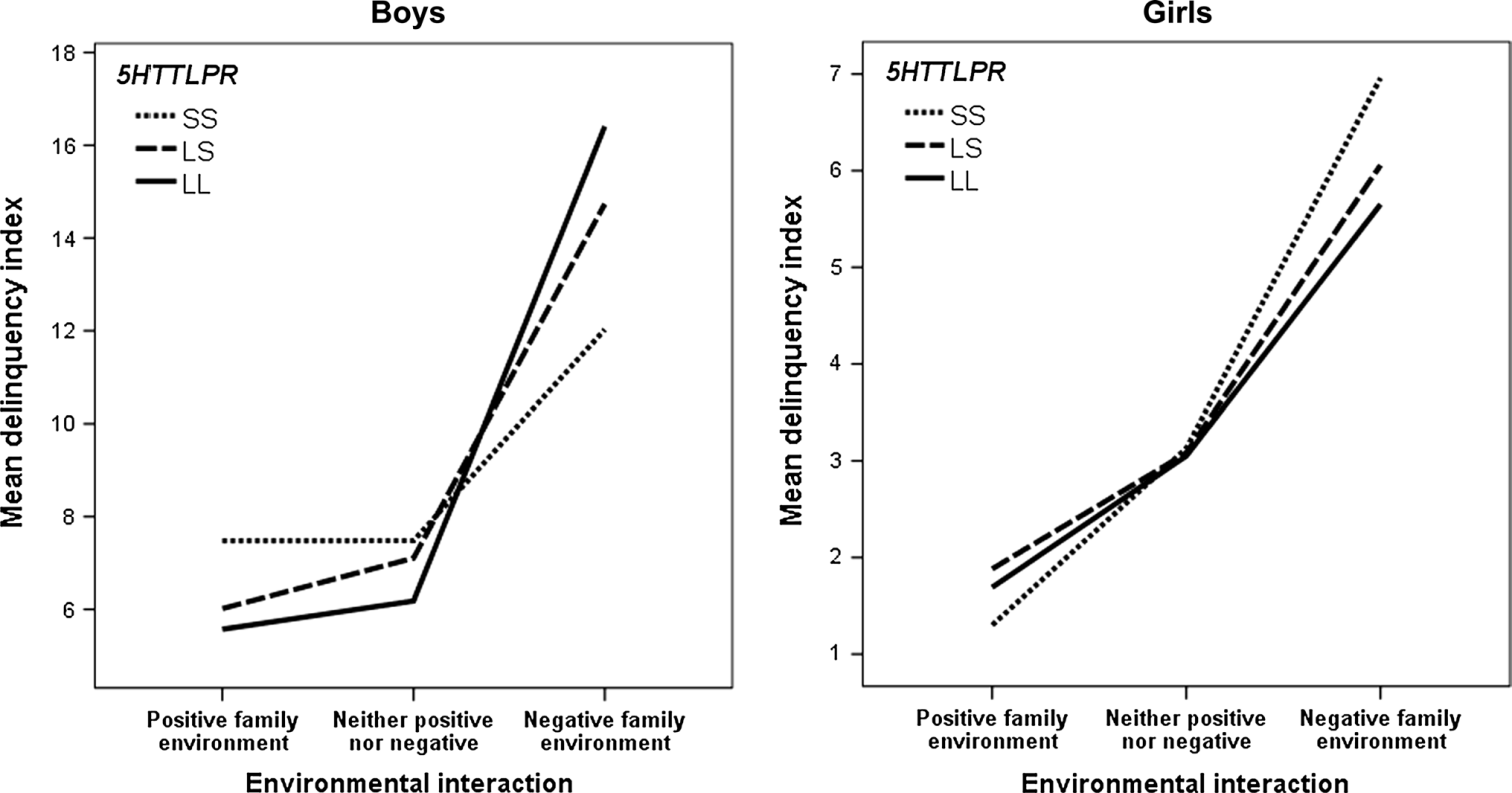
Interaction between 5HTTLPR and positive and negative family environments in relation to delinquency among boys and girls

For illustrative purposes (Fig. 2), a variable combining the family adversity and positive family relations indices was created as follows: *Positive family environment*: no family adversity (0 points) and positive family relations (highest quartile, Q4) (*n* = 284); *Neither positive nor negative*: no family adversity (0 points) and no positive family relations (Q1–Q3) or family adversity (≥ 1 point) and positive family relations (Q4) (*n* = 821); *Negative family environment*: family adversity (≥ 1 point) and no positive family relations (Q1–Q3) (*n* = 423).

*Symptoms of depression* The participants were asked to indicate whether any of the following seven core symptoms of depression from the Depression Self-Rating Scale (DSRS) (Svanborg and Ekselius 2003) had occurred during the last 2 weeks: dysphoric mood, loss of interest or pleasure in most activities, irritated mood/feeling upset all the time, feelings of worthlessness or guilt, concentration disturbances, fatigue or loss of energy, and suicidal thoughts. Questions of somatic symptoms, weight loss, and sleep disturbances were omitted to identify core symptoms of depressive mood (Hieronymus et al. 2016; Trivedi et al. 2004). The internal consistency of the symptoms of depression items measured by Cronbach’s *α* was 0.813.

*Delinquency* Delinquency was measured using the following questions: How often have you… (1) Taken goods in a store, shop, or kiosk without paying? (2) Deliberately smashed or wrecked windows, streetlights, benches, or other public things? (3) Taken money at home that did not belong to you? (4) Painted graffiti, or scrawled on, for example, a public wall, without permission? (5) Sold or bought something you knew was stolen? (6) Stolen a bike? (7) Dodged payment at the movies or a café, on a bus, train, or similar? (8) Driven a moped, motorbike, or car while drunk? (9) Broken into a house, shop, store, kiosk, or other building with the intention to steal things? (10) Stolen anything from another person’s pocket or bag? (11) Threatened or forced someone to give you money, cigarettes, or anything else? (12) Stolen a car? (13) Stolen a moped, motorbike, or motor scooter? (14) Been involved in breaking into and stealing something from a car? (15) By yourself broken into and stolen something from a car? (16) Been involved in a fight during your leisure time (not at school)? (17) Been involved in threatening another person to do something he/she did not want to? (18) By yourself threatened another person to do something he/she did not want to? (19) Carried a weapon (knuckle-duster, baseball bat, knife, switchblade, or similar) at school or during your leisure time? (20) Hit/kicked someone so hard he/she needed medical attention? (21) Deliberately hurt someone with a knife, switchblade, knuckle-duster, or similar?

The response alternatives were: never (0), once (1), 2–4 times (2), 5–10 times (3), and more than 10 times (4). A delinquency index was created as a summation of questions 1–21 (range 0–84 points), as described previously (Åslund et al. 2011). The internal consistency of the delinquency items measured by Cronbach’s *α* was 0.907.

### Sample 2: SALVe 2001

#### Participants and procedure

In 2001, 4260 adolescents (16 and 19 years old) were asked to complete the Survey of Adolescent Life in Västmanland (SALVe) questionnaire during a 1-h classroom session, supervised by a specially trained research assistant. All students had the opportunity to give their informed consent to participate in an in-depth interview and the drawing of a blood sample, by including their full personal security number on the form’s front page. Informed consent was received from 785 students. They were classified according to a risk index, depending on behaviours reported in the questionnaire (alcohol and drug risk behaviour, sexual risk behaviour, property, and violent offence). Randomized samples of 400 students matched for age, sex, and risk behaviour were drawn. The procedure using the initial risk index ensured enough participants were recruited from each end of the deviant behaviour continuum. Eighty-one boys and 119 girls took part in an interview in 2001 and provided blood samples for DNA extraction. The study sample has been previously described elsewhere (Nilsson et al. 2006).

#### Measures

Sex and ethnicity were classified in the same way as for the 2006 sample.

*Positive family relations* The participants were asked about their relationship to their parents in the first classroom questionnaire: (1) Do you feel close to your parents? (2) Do you like to be with your parents? (3) Do you share your feelings with your parents? (4) Do your parents give you many opportunities to do fun things with them? (5) Do your parents ask for your opinion before making decisions that affect you? (6) Do your parents notice when you have done something good and praise you? Answer alternatives were: no, neither of my parents (0); yes, one of my parents (1); and yes, both of my parents (2). A summation index of these questions was created, with a possible score range of 0–12. The internal consistency of the parent–child relationship items measured by Cronbach’s *α* was 0.792.

*Physical and sexual abuse* In the interview, the participants were asked to describe different experiences of negative things that they could have been exposed to. If no significant maltreatment or abusive experiences were detected, a more direct question was asked: “Have you ever been exposed to any type of mean treatment from others?” and followed by examples such as; being hit, or exposed to shameful or disgraceful handling or sexually abusive activities by others. This topic was coded “experiences of maltreatment/abuse/sexual abuse”, with classifications of Yes or No. This transformation of answers from the research interview of the psychosocial factor was made to mimic a clinical interview pertaining to Axis IV: Psychosocial and Environmental Problems of the Diagnostic and Statistical Manual of Mental Disorders (DSM-IV), which is usually considered a useful instrument for organizing and communicating clinical information (APA 2000). Inter-rater reliability (measured as Cohen’s *κ*) for two raters, who listened to a 10% sample of the audiotaped interviews, was 1.0. The measure has been described previously (Nilsson et al. 2008).

*Symptoms of depression* This was measured as in the SALVe 2006 sample. The internal consistency of the depression symptom items measured by Cronbach’s *α* was 0.831.

*Delinquency* The following questions were applied in the interview to measure delinquency: How often have you (1) Fought in school? (2) Fought during your leisure time? (3) Hit/kicked someone so that he/she needed medical care? (4) Carried a knife, knuckle-duster, or other weapon? (5) Hurt someone with a knife, knuckle-duster, or other weapon? (6) Threatened or forced someone to give you money, cigarettes, or something else? (7) Threatened or forced someone to do something he/she did not want to do? (8) Shoplifted (CDs, clothes, make-up, or other things)? (9) Taken money that did not belong to you? (10) Taken mobile phones, clothes, bicycles, mopeds, or other things that did not belong to you? (11) Broken into cars, boats, cellars, houses, or similar? (12) Smashed or wrecked windows, street-lamps, benches, or other things? (13) Painted graffiti, scrawled, or similar? (14) Destroyed things in school? (15) Driven a motor vehicle while drunk?

Positive responses to any of these questions were followed by questions regarding time and place (when, where, and with whom), frequency, and other relevant circumstances. The results from the questions were classified as: no (0); yes, infrequently (1); yes, frequently (2); yes, frequently and continuing (3). A summation index of the above questions was created for total delinquency, with a possible score range of 0–45 (Nilsson et al. 2014). The internal consistency of the delinquency items measured by Cronbach’s *α* was 0.830.

### Genetic analyses

The serotonin transporter-linked promoter region (5HTTLPR) polymorphism of the *SLC6A4* gene consists of an insertion/deletion that creates a short (S) 14 repeat or a long (L) 16 repeat allele, and was analysed as described previously (Åslund et al. 2009; Sjoberg et al. 2006). The 5HTTLPR allelic distribution was in Hardy–Weinberg equilibrium (*p* = 0.20, and 0.21 for SALVe 2006 and SALVe 2001, respectively). In the present study, homozygous individuals for the 14 repeat allele of the 5HTTLPR were coded as SS, heterozygous individuals for the 14 and 16 repeat alleles were coded as LS, and homozygous individuals for the 16 repeat allele were coded as LL.

### Ethics

The studies were approved by the Regional Ethical Review Board of Uppsala, Dr.nr 00-325 and 2005:375, and carried out following the rules of the Declaration of Helsinki of 1964 revised in 2008. Verbal and/or written informed consent was obtained from the participants.

### Statistical analysis

Main and interaction effects of genetic and environmental factors were analysed by general linear models (GLMs). GLMs were validated using Poisson regression. A *p* value < 0.05 was considered significant for main effects, and *p* < 0.10 was considered significant for interaction effects (Fleiss 1986). A model-dependent realistic analysis (Hawking and Mlodinow 2010) was performed, taking possible sex-specific genetic susceptibility into account. Analyses were based on the assumption that associations of a genotype with the outcome would change when sex or a positive or negative environmental factor was entered into the statistical model. Sex differences, differences between late-responders and the total study population, and differences between participants with available 5HTTLPR data and those with missing 5HTTLPR data were analysed by Chi-square analysis and Mann–Whitney *U* test.

## Results

Distributions of the study samples are presented as categorical and continuous data in Table 1.

**Table 1:** Demographic data for the two samples

Nominal and dichotomized variables	Total		Boys		Girls		*χ* ^2^	*p*
	*n*	%	*n*	%	*n*	%		
*SALVe 2006*								
Sex	1457	100.0	743	51.0	714	49.0	0.58	0.447
*5HTTLPR*								
SS	304	20.9	147	19.8	157	22.0		
LS	695	47.7	366	49.3	329	46.1		
LL	458	31.4	230	31.0	228	31.9	1.73	0.421
Non-scandinavian ethnicity	211	14.5	116	15.6	95	13.3	1.57	0.211
*SALVe 2001*								
Sex	191	100.0	79	41.4	112	58.6	5.70	0.017
*5HTTLPR*								
SS	45	23.6	19	24.1	26	23.2		
LS	86	45.0	29	36.7	57	50.9		
LL	60	31.4	31	39.2	29	25.9	4.71	0.095
Non-scandinavian ethnicity	26	13.6	15	19.0	11	9.8	3.31	0.069
Physically and/or sexually abused	37	19.4	13	16.5	24	21.4		
Not abused	154	80.6	66	83.5	88	78.6	0.733	0.392

Ordinal and index variables	M	SD	M	SD	M	SD	*Z*	*p*
SALVe 2006 (*n* = 1457)								
Family adversity	0.52	0.93	0.42	0.83	0.62	1.01	− 3.84	< 0.001
Positive family relations	12.12	3.08	11.98	3.09	12.27	3.06	− 2.22	0.026
Family subjective SES	4.30	1.03	4.36	1.06	4.25	1.01	− 2.16	0.031
Symptoms of depression	1.72	2.04	1.29	1.79	2.18	2.18	− 8.69	< 0.001
Delinquency	6.29	9.60	8.74	11.82	3.75	5.48	− 9.02	< 0.001
SALVe 2001 (*n* = 191)								
Positive family relations	8.49	3.12	8.68	3.42	8.36	2.90	− 1.41	0.158
Symptoms of depression	1.68	1.96	1.08	1.74	2.10	2.00	− 4.035	< 0.001
Delinquency	4.52	5.70	6.60	6.61	3.03	4.41	− 4.78	< 0.001

Sex differences are analysed with Chi square test and Mann–Whitney *U* test

*SES* socio-economic status

In the total SALVe 2006 sample (*n* = 1457), there was a correlation between depression and delinquency (*ρ* = 0.161, *p* < 0.001), and this was somewhat stronger when analysed for boys and girls separately (boys; *ρ* = 0.229, *p* < 0.001; girls; *ρ* = 0.234, *p* < 0.001). In the SALVe 2001 sample (*n* = 191), no such correlation was found (*ρ* = − 0.027, *p* = 0.771).

### Differential susceptibility effects in relation to depressive symptoms

Table 2 shows the results for the 5HTTLPR interaction effects with the positive and negative environmental factors in relation to depressive symptoms in the larger SALVe 2006 sample. There was a three-way interaction of the 5HTTLPR and both positive and negative life events in both samples, implying that both positive and negative environmental factors interacted with the 5HTTLPR in relation to depressive symptoms. In addition, when the sex interaction term was included in the model, the sex × 5HTTLPR × family adversity × positive family relations interaction was significant in both the GLM and the Poisson model, and the eta-square was also stronger. In a multivariable model with the same variables that excluded all interaction terms (Adj. *R*^2^ = 0.216), all variables except for 5HTTLPR (*p* = 0.057) showed significant effects on depressive symptoms. This indicates that all environmental factors were of importance, although the strength of the model increased when adjusted for interactions with the 5HTTLPR.

**Table 2:** General linear models and Poisson regression models of symptoms of depression for the SALVe 2006 sample (*n* = 1457)

SALVe 2006	GLM			Poisson regression	
	*F*	*p*	*η* ^2^	Wald *χ*^2^	*p*
Model 1^a,b^					
Sex	90.370	< 0.001	0.069	163.395	< 0.001
*5HTTLPR*	0.542	0.582	0.001	6.432	0.040
Family adversity	7.271	< 0.001	0.034	0.007	0.935
Positive family relations	2.271	0.003	0.029	75.822	< 0.001
*5HTTLPR* × family adversity	1.856	0.055	0.013	7.341	0.025
*5HTTLPR* × positive family relations	1.717	0.011	0.039	7.226	0.027
*5HTTLPR* × family adversity × positive family relations	1.533	0.001	0.114	16.913	0.001
	Adj. *R*^2^ = 0.256				
Model 2^a,c^					
Sex	2.449	0.118	0.002	13.432	< 0.001
*5HTTLPR*	1.110	0.330	0.002	8.643	0.013
Family adversity	6.003	< 0.001	0.031	0.517	0.472
Positive family relations	2.287	0.003	0.031	69.562	< 0.001
Sex × *5HTTLPR*	1.189	0.305	0.002	4.462	0.107
Sex × *5HTTLPR* × family adversity	1.860	0.012	0.032	11.996	0.035
Sex × *5HTTLPR* × positive family relations	1.521	0.005	0.084	11.909	0.036
Sex × *5HTTLPR* × family adversity × positive family relations	1.270	0.022	0.142	19.538	0.003
	Adj. *R*^2^ = 0.259				

^a^Adjusted for ethnicity, family subjective socio-economic status, and delinquency

^b^Model 1 presenting no adjustment for sex interaction effects

^c^Model 2 including the sex interaction effects

Table 3 shows the results for the 5HTTLPR interaction effects with the positive and negative environmental factors in relation to depressive symptoms in the smaller SALVe 2001 sample. Similar to the findings in the SALVe 2006 sample (Table 2), there was a three-way interaction of the 5HTTLPR and both the positive and the negative environmental factors, implying that both positive and negative environmental factors interacted with 5HTTLPR in relation to depressive symptoms. However, when the sex interaction variable was included in the model, the sex × 5HTTLPR × physical and sexual abuse × positive family relations’ interaction was only borderline significant in the GLM model, although it was significant in the Poisson regression model. Furthermore, in both models, the cG × E interaction models showed a stronger interaction effect for the positive environmental factors than the negative environmental factors.

**Table 3:** General linear models and Poisson regression models of symptoms of depression for the SALVe 2001 sample (*n* = 191)

SALVe 2001	GLM			Poisson regression	
	*F*	*p*	*η* ^2^	Wald *χ*^2^	*p*
Model 1^a,b^					
Sex	11.662	0.001	0.080	34.023	< 0.001
*5HTTLPR*	0.217	0.805	0.003	8.058	0.018
Physical and sexual abuse	5.013	0.027	0.036	0.144	0.704
Positive family relations	1.366	0.190	0.108	0.732	0.392
*5HTTLPR* × physical and sexual abuse	1.248	0.290	0.018	4.529	0.104
*5HTTLPR* × positive family relations	1.698	0.041	0.201	4.573	0.102
*5HTTLPR* × physical and sexual abuse × positive family relations	1.747	0.045	0.172	17.328	0.001
	Adj. *R*^2^ = 0.206				
Model 2^a,c^					
Sex	9.309	0.003	0.076	2.157	0.142
*5HTTLPR*	1.209	0.302	0.021	5.141	0.077
Physical and sexual abuse	1.111	0.294	0.010	0.280	0.597
Positive family relations	1.230	0.271	0.116	2.759	0.097
Sex × *5HTTLPR* × physical and sexual abuse	0.418	0.795	0.015	5.651	0.342
Sex × *5HTTLPR* × positive family relations	1.235	0.196	0.299	5.749	0.219
Sex × *5HTTLPR* × physical and sexual abuse × positive family relations	1.543	0.107	0.160	13.475	0.019
	Adj. *R*^2^ = 0.162				

^a^Adjusted for ethnicity

^b^Model 1 presenting no adjustment for sex interaction effects

^c^Model 2 including the sex interaction effects

### Differential susceptibility effects in relation to delinquency

Table 4 shows the results for the 5HTTLPR interaction effects with the positive and negative environmental factors in relation to delinquency in the larger SALVe 2006 sample. Table 5 shows the same analyses on the smaller SALVe 2001 sample. The models investigating delinquency were much stronger than the models investigating depressive symptoms. All tested interactions were significant in the SALVe 2006 sample using two different statistical methods, and when excluding and including the sex interaction terms. Nearly all tested interactions were similarly significant in the SALVe 2001 sample. There was a three-way interaction of the 5HTTLPR and both positive and negative life events in both samples, implying that both positive and negative environmental factors interacted with the 5HTTLPR in relation to delinquency. Furthermore, according to the eta-square, there was a stronger interaction effect for positive family relations than the negative environmental factors in both samples. The main effects varied in strength depending on the interaction terms inserted into the models. For example, in the SALVe 2006 sample, the main effect of sex was not significant in the Poisson regression model that included sex interaction effects. In a multivariable model with the same variables that excluded all interaction terms (Adj. *R*^2^ = 0.184), sex and family adversity were significant (*p* < 0.001), whereas positive family relations (*p* = 0.139) and 5HTTLPR (*p* = 0.658) were not.

**Table 4:** General linear models and Poisson regression models of delinquency for the SALVe 2006 sample (*n* = 1457)

SALVe 2006	GLM			Poisson regression	
	*F*	*p*	*η* ^2^	Wald *χ*^2^	*p*
Model 1^a,b^					
Sex	122.726	< 0.001	0.088	1687.147	< 0.001
*5HTTLPR*	0.257	0.773	< 0.001	39.542	< 0.001
Family adversity	7.653	< 0.001	0.035	0.008	0.929
Positive family relations	2.707	< 0.001	0.033	106.757	< 0.001
*5HTTLPR* × family adversity	6.495	< 0.001	0.044	34.970	< 0.001
*5HTTLPR* × positive family relations	4.095	< 0.001	0.088	39.662	< 0.001
*5HTTLPR* × family adversity × positive family relations	2.370	< 0.001	0.166	131.926	< 0.001
	Adj. *R*^2^ = 0.296				
Model 2^a,c^					
Sex	73.587	< 0.001	0.059	0.021	0.886
*5HTTLPR*	0.421	0.657	0.001	15.007	0.001
Family adversity	8.207	< 0.001	0.040	4.752	0.029
Positive family relations	1.747	0.033	0.023	174.902	< 0.001
Sex × *5HTTLPR*	3.449	0.032	0.006	22.629	< 0.001
Sex × *5HTTLPR* × family adversity	4.197	< 0.001	0.073	70.346	< 0.001
Sex × *5HTTLPR* × positive family relations	2.529	< 0.001	0.131	93.902	< 0.001
Sex × *5HTTLPR* × family adversity × positive family relations	1.721	< 0.001	0.179	177.451	< 0.001
	Adj. *R*^2^ = 0.308				

^a^Adjusted for ethnicity, family subjective socio-economic status, and core symptoms of depression

^b^Model 1 presenting no adjustment for sex interaction effects

^c^Model 2 including the sex interaction effects

**Table 5:** General linear models and Poisson regression models of delinquency for the SALVe 2001 sample (*n* = 187)

SALVe 2001	GLM			Poisson regression	
	*F*	*p*	*η* ^2^	Wald *χ*^2^	*p*
Model 1^a,b^					
Sex	8.924	0.003	0.064	131.635	< 0.001
*5HTTLPR*	0.946	0.391	0.014	18.372	< 0.001
Physical and sexual abuse	8.757	0.004	0.063	1.254	0.263
Positive family relations	2.602	0.004	0.192	8.769	0.003
*5HTTLPR* × physical and sexual abuse	4.727	0.010	0.067	11.208	0.004
*5HTTLPR* × positive family relations	2.015	0.010	0.235	18.039	< 0.001
*5HTTLPR* × physical and sexual abuse × positive family relations	1.968	0.020	0.194	2.086	0.555
	Adj. *R*^2^ = 0.358				
Model 2^a,c^					
Sex	14.047	< 0.001	0.114	30.204	< 0.001
*5HTTLPR*	1.669	0.193	0.030	21.697	< 0.001
Physical and sexual abuse	14.075	< 0.001	0.114	0.040	0.841
Positive family relations	2.118	0.021	0.189	2.795	0.095
Sex × *5HTTLPR* × physical and sexual abuse	2.263	0.067	0.077	18.986	0.001
Sex × *5HTTLPR* × positive family relations	1.635	0.025	0.369	37.860	< 0.001
Sex × *5HTTLPR* × physical and sexual abuse × positive family relations	2.827	0.001	0.266	7.856	0.097
	Adj. *R*^2^ = 0.392				

^a^Adjusted for ethnicity

^b^Model 1 presenting no adjustment for sex interaction effects

^c^Model 2 including the sex interaction effects

To illustrate the sex differences in the cG × E interaction models statistically expressed in the tables, separate figures for boys and girls are presented on delinquency for the SALVe 2006 sample (Fig. 2). However, note that a three- or four-way interaction cannot be expressed in a two-dimensional figure, and the figures, therefore, only serve an illustrative purpose. Figure 2 illustrates that the strongest susceptibility properties in males were among individuals homozygous for the long 5HTTLPR allele, whereas the strongest susceptibility properties in females were among individuals homozygous for the short 5HTTLPR allele.

In the SALVe 2006 sample, late responders reported slightly higher scores on the delinquency measure compared to the total sample (*Z* = − 3.082, *p* = 0.002). No differences for depressive symptoms, family adversity, or positive family relations were found among late responders compared to the total sample (data not shown). When comparing participants with available 5HTTLPR to those with missing 5HTTLPR data, no differences were found for delinquency, family adversity, or positive family relations (data not shown). However, participants with missing 5HTTLPR data reported slightly lower scores of depressive symptoms (*Z* = − 2.135, *p* = 0.003). This finding may be explained by a higher rate of missing 5HTTLPR data among boys (*χ*^2^ = 11.001, *p* = 0.001).

## Discussion

In the present study, we aimed to test whether (a) there was a differential susceptibility effect of the 5HTTLPR in relation to the emotion regulated phenotypes of depressive symptoms and delinquency, and (b) whether the possible susceptibility properties of the 5HTTLPR differed between the sexes.

We found support for differential susceptibility properties of the 5HTTLPR in relation to both depressive symptoms and delinquency in two independent adolescent samples, in that the direction of the genetic effect depended on both positive and negative environmental exposure. However, support for the differential susceptibility properties of the 5HTTLPR was stronger in relation to delinquency than to depressive symptoms. Moreover, the results suggested sex differences regarding the susceptibility properties of the 5HTTLPR. The strongest susceptibility effects in males were illustrated for the individuals homozygous for the L allele of the 5HTTLPR, while the strongest susceptibility effects in females were illustrated for the individuals homozygous for the S allele in relation to delinquency.

The findings support the theories of differential susceptibility (Belsky and Pluess 2009) and biological sensitivity to context (Boyce and Ellis 2005), which, in contrast to the traditional diathesis–stress framework, suggests that cGs that interact with environmental events do not exclusively confer a risk for behavioural or psychiatric disorders but rather seem to alter the sensitivity to both positive and negative influences in the environment (Belsky and Beaver 2011; Belsky et al. 2009; Belsky and Pluess 2009; Hankin et al. 2011). Carriers of plasticity alleles who are raised in a positive environment show better-than-average positive outcomes, whereas carriers of the same alleles raised in adverse conditions show negative outcomes, compared with non-carriers (Reiss et al. 2013; Hankin et al. 2011; Nilsson et al. 2014). The present study suggests such susceptibility properties of the 5HTTLPR in relation to depressive symptoms and delinquent behaviour, of which both are phenotypical outcomes that are closely related to emotion regulation mechanisms. Moreover, in both samples, the cG × E interaction models showed a stronger interaction effect for positive environmental factors than negative environmental factors, further emphasizing the importance of applying a differential susceptibility perspective in future cG × E investigations of the 5HTTLPR.

Several previous cG × E models of depression have reported weak effect sizes (Culverhouse et al. 2017), whereas cG × E models of aggressive behaviour or delinquency often report stronger effect sizes, in line with the two original cG × E studies by Caspi et al. (2002, 2003). It has been suggested that the impact of the 5HTT on behaviour may be broader than is commonly appreciated and that it may play a role in social cognition (Canli and Lesch 2007). If we presume that the most common first primal reaction to emotional stress is aggression, models of intersecting phenotypes such as delinquency, antisocial behaviour, etc, will generate distinct associations. Composite evidence suggests that the 5HTTLPR is related to the aetiology of different mental illnesses, although it has been stressed that age composition and sex composition of the samples along with operationalization of the phenotypic and environmental exposures are important when studying different phenotypes (Uher and McGuffin 2008). We suggest that the 5HTTLPR polymorphism, presumably in addition to other related genetic variations within the monoaminergic system (Iofrida et al. 2014), has a general impact on the social emotion regulations system, which in different studies may be conceptualized as, for example, impulsivity (Paaver et al. 2007), anxiety (Munafo et al. 2005a), depression (Sharpley et al. 2014; Bleys et al. 2018; Culverhouse et al. 2017), alcohol consumption (Nilsson et al. 2005; Munafo et al. 2005b), eating disorders (Rozenblat et al. 2017), and aggressive and antisocial behaviour (Ficks and Waldman 2014; Iofrida et al. 2014).

We used a model-dependent realistic analysis (Hawking and Mlodinow 2010) and investigated the 5HTTLPR × environment in relation to depression and delinquency, and whether the directions of cG × E varied depending on both sex and the different environmental exposures (Comasco et al. 2013; Reiss et al. 2013). Because there is no solid knowledge about the phenotypical susceptibility properties of the 5HTTLPR, or about different combinations of positive and negative environmental exposures, a directional hypothesis-driven method could not be used. The model-dependent realistic analysis differs from the belief-dependent realism that relies on reference anchors or small pieces of information, and emphasizes seeking and confirming evidence to support the existing beliefs (Shermer 2011). When the sex, genetic, and environmental factors are truly interacting, a statistical model will constantly change depending on which factors and interaction terms are included in the analysis. It is, therefore, always important to include the relevant interaction terms in the applied models.

In our view, conclusions cannot be drawn about genetic–phenotypic associations if the gene investigated has susceptibility properties, and the samples are not investigated with regard to both positive and negative environmental factors. Moreover, if there is a sex-dependent genetic interaction of the environment in relation to the phenotypes studied, the main effect of sex as well as the interaction terms of sex should be included in the models to adjust for that effect. Consequently, in the case of sex differences, sex-separated analyses will not show the full properties of the cG × E interaction studied.

In the present study, we used core symptoms of depressive mood to measure symptoms of depression. A study investigating factor loadings of four commonly used depression scales (QIEDS-C, QIDS-SR, IDS-C, and IDS-SR) revealed the highest item-total correlations for core depressive symptoms including sad mood, involvement, energy, concentration, and self-outlook (Trivedi et al. 2004). Moreover, these items were most accurately associated with the capacity to detect change in overall symptom severity following anti-depressant treatment (Trivedi et al. 2004). Another study found that somatic symptoms such as weight disturbances and sexual functioning showed low and/or non-significant effect sizes in relation to the effect of anti-depressant medication (Hieronymus et al. 2016). Those authors suggested that focusing on the core symptoms of depressive mood might be a more accurate method of measuring depression (Hieronymus et al. 2016), omitting somatic items such as sleep disturbances, psychomotor disturbances, weight disturbances, and general somatic symptoms, which are ambivalent and multifactorial symptoms common in a vast number of diseases. Moreover, these factors are common side effects of many anti-depressive medications. The inclusion of somatic items in measures of depressive symptoms may thereby decrease the sensitivity of the measurement (Hieronymus et al. 2016). In the present study, core symptoms of depression are, therefore, defined by a summation index of items from the DSRS DSM-IV scale (Svanborg and Ekselius 2003), including dysphoric mood, loss of interest or pleasure in most activities, irritated mood/feeling upset all the time, feelings of worthlessness or guilt, concentration disturbances, fatigue or loss of energy, and suicidal thoughts.

### Sex differences in cG × E effects and possible evolutionary aspects

It has been suggested that the serotonin system behaves differently in males and females (Biver et al. 1996; Costes et al. 2005; Williams et al. 2003; Brummet et al. 2008; Walderhaug et al. 2007). Moreover, we and others have previously reported sex differences of the 5HTTLPR detected by cG × E analyses that were carried out separately within each sex (Sjoberg et al. 2006; Åslund et al. 2009, 2013; Brummett et al. 2008a). However, such analyses only show different directions of the findings in relation to sex and do not allow for testing whether those differences in direction are statistically significant. To analyse sex differences statistically, both the main and interaction terms of sex and the genes of interest must be included in the same model. Moreover, since the sex × gene effect in relation to the phenotypes of interest is an important part of the evolutionary assumption on which the analyses have been made, sex-separated analyses would be erroneous.

Evolutionary theories of genetic diversity state that there is an evolutionary advantage to choosing genetically dissimilar mates (Fromhage et al. 2009). The main benefit is the production of offspring with optimally dissimilar alleles across many loci, providing a wider range of potentially useful gene products to allow better preparation for the unpredictable interactions and environmental changes faced in the next generation. This increases the chances that offspring will adapt to different environmental conditions (Brown 1997; Mays and Hill 2004). Another benefit is to increase the heterozygosity of the offspring, which might benefit individuals by the masking of lethal or sub-lethal alleles or by producing an over dominance for fitness where the heterozygote is more fit than either of the homozygotes (Brown 1997). Both laboratory and field studies of different organisms, including humans, have provided empirical evidence for the choice of genetically dissimilar mates (Mays and Hill 2004; Wedekind and Füri 1997; Wedekind et al. 1995; Chaix et al. 2008).

From an evolutionary biological perspective, phenotypic selection in different species is often distinct in the two sexes, leading to genetic conflict over sex-specific phenotypic optima, where changes in allele frequency at loci confer a fitness advantage to one sex while at the same time conferring costs to the other (Berger et al. 2014; Bonduriansky and Chenoweth 2009; Rice 1984). Such intralocus sexual conflict has been suggested to affect fundamental evolutionary processes, i e., maintenance of genetic variation (Arnqvist 2011; Fry 2010), differentiation between populations (Connallon and Clark 2012; Hesketh et al. 2013), and adaptation to environmental change (Whitlock and Agrawal 2009; Berger et al. 2014). Sexual antagonism can be resolved through the evolution of sex-specific gene expression, allowing the sexes to diverge phenotypically (Hesketh et al. 2013; Arnqvist 2011). Thereby, it has been predicted that a large proportion of the standing genetic variation in fitness will have opposite effects in males and females in well-adapted populations (Berger et al. 2014; Arnqvist 2011). Evolutionary intralocus sexual conflict might thus be a possible explanation to sex-differentiated genetic expression of specific candidate genes that have had a major evolutionary influence on reproductive success and survival in humans (Berger et al. 2014; Mank 2017). Phenotypic expressions associated with serotonergic function, such as sexual behaviour, sensorimotor reactivity, social cognition, and emotion-related behaviour and regulation, are presumably factors of such evolutionary importance.

### Limitations and strengths

The findings of the present study must be interpreted in light of the study’s limitations. First, the SALVe 2001 sample was small and consequently has low power. However, the explained variances of the statistical models were acceptable in both samples, with a range of 0.16–0.25 for the analyses of depressive symptoms and 0.30–0.39 for the analyses of delinquency. The low power also resulted in the lack of adjustment for possible confounding from depressive symptoms in the delinquency model and vice versa in the SALVe 2001 sample, as well as the lack of adjustment for the sex × 5HTTLPR interaction. Moreover, no data on socio-economic status were available in the SALVe 2001 sample.

Second, the importance of considering adjustment for multiple testing must be discussed. In the present study, the hypotheses were analysed in four models, giving a Bonferroni corrected *p* value of 0.0125. However, we also controlled our models with complementary statistics, giving a second Bonferroni corrected *p* value of 0.006. In addition, we performed a series of univariable analyses in the descriptive section, giving a third Bonferroni corrected *p* value of 0.0023. The present study illustrates the negative side effects of adjustment for multiple testing. In the smaller sample, some of the significant results should be excluded according to Bonferroni adjustment. However, studies with smaller sample sizes often have more carefully investigated subjects, and also stronger effect sizes estimated by *R*^2^ and *η*^2^ values, such as those shown in our study. Smaller samples suffer from extensive adjustment for multiple testing, whereas larger samples automatically get better *p* values, despite weaker models. Thereby, the use of extensive multiple testing procedures and judging the results by the *p* values instead of the model effect sizes might increase the risk of Type II errors (Perneger 1998; Nakagawa 2004). However, compared to the actual empirical *p* values, and depending on the level of Bonferroni adjustment chosen, the findings of the present study were significant. We believe that our results are solid, because the results were replicated in two separate samples, and using complementary statistics. In future cG × E studies, we advocate using complementary statistics and replications in different samples, as well as including different types of measurements of both the dependent and independent variables; if significant, these will indicate solid models.

Third, both the depressive symptoms variable and the delinquency variable were skewed and neither a log- nor a log–log transformation produced a symmetric distribution of the data. It is difficult to choose appropriate statistical methods in a study of cG × E where the outcome measures are on skewed ordinal or interval scales. While interactions are efficiently estimated by a GLM, the inference is not valid if the assumption of normally distributed residuals is violated. Therefore, although GLMs could be used to estimate main and interaction effects, the results should be interpreted with caution. However, we confirmed the GLM results with complementary Poisson log-linear regressions, which are suitable for ordinal scaled variables and the kinds of distributions that were found in the present study. Procedures with complementary statistical approaches can help to overcome shortcomings of individual statistical methods and help to eliminate scaling artefacts, one of the ubiquitous sources of artefacts in interaction research.

Fourth, we reached only 77.4% of the target population in SALVe 2006, which may have influenced the results. There were 183 late responders, who completed the questionnaire at a later time because of their absence from school at the day of the survey. No significant differences for depressive symptoms, family adversity, or positive family relations were found in the late respondent group compared with the total population. However, the late respondents reported slightly higher delinquency scores. According to Miller and Smith (Miller and Smith 1983), late respondents tend to be similar to non-respondents in survey studies. Thus, the mean delinquency might have been somewhat higher if non-responders had been included in the study, possibly influencing the results. Due to problems with 5HTTLPR analyses, 586 participants were excluded from the SALVe 2006. The DNA extraction method was not optimal and, in some cases, yielded poor quality DNA. The anonymous design did not allow us to repeat the DNA collection. When comparing participants with available 5HTTLPR data with those who had missing data, there were no differences in delinquency, family adversity, or positive family relations, although participants with missing 5HTTLPR data reported lower scores on depressive symptoms. This might be explained by a higher rate of missing 5HTTLPR data among boys.

Fifth, in the SALVe 2001 sample, the LL allelic frequency was lower than expected among girls. The 5HTTLPR SS has been suggested to be present in 22% of Caucasians and in 60% of Asians, whereas the LL is present in 29–43% of Caucasians, and in 1–13% of East Asians (Iurescia et al. 2016). In the present study, the genetic distribution was in Hardy–Weinberg equilibrium. Nevertheless, this is a small sample and thereby more sensitive to bias. On the other hand, there were no significant differences in allelic frequency, neither between boys and girls, nor between girls in the two samples. Moreover, the single-nucleotide polymorphism rs25531 and the triallelic nature of the 5HTTLPR were not analysed in the present study. This triallelic nature has been described as an A > G polymorphism at position 6 of the first of two 22-bp imperfect repeats defining the 16-repeat L allele and has been suggested to be equivalent in expression to the S allele (Nakamura et al. 2000). The L_G_ allele has been reported to have a prevalence of 9–14% in Caucasians and 24% in African-Americans, and the three alleles (S, L_A_, and L_G_) have been suggested to act differently (Hu et al. 2005). Another A > G substitution has been identified in the S allele (Kraft et al. 2005). Thereafter, a study showed that rs25531 lies 18 bp 5′ to the site where the 43 bp insertion/deletion defines the 14- and 16-repeat alleles of the 5HTTLPR and suggested that these polymorphisms should be considered as four alleles instead of a triallelic unique locus (Perroud et al. 2010). The rs25531 has shown altered binding of nuclear extracts to a sequence for the activator protein 2 (AP-2) transcription factor, which is believed to be a critical factor in regulating neural gene expression in mammals (Kraft et al. 2005). Furthermore, rs25531 has revealed evidence of an association with antidepressant treatment response of selective serotonin reuptake inhibitors (SSRIs) (Perroud et al. 2010; Kraft et al. 2005). Among SSRI non-responders, the long 5HTTLPR allele is found more often than anticipated, given that the minor rs25531 G allele is present (Kraft et al. 2005) and the very rare G-14/G-16 polymorphism has been identified in a small sample of women with a history of suicide attempt and borderline personality disorder (Perroud et al. 2010). On one hand, we acknowledge rs25531 as an important factor that increase the genetic variability of the *SLC6A4* promoter, and therefore, the failure to adjust for rs25531 is a major limitation of the study.

On the other hand, recent evidence suggests that 5HTTLPR contains at least 28 different repetitive units, and the 5HTTLPR shows 40 different allelic variants currently known (Iurescia et al. 2016). Further factors influencing the *SLC6A4* expression are, for example, intronic and 3′-UTR variability and epigenetic mechanisms (Iurescia et al. 2017), which are beyond the scope of the present report. Future studies will thus need to elucidate the functional relevance of these allelic variants for the regulation of *SLC6A4* expression.

Finally, we did not have any information on present or past diagnoses or treatment. The results are solely based on self-reported depressive symptoms and delinquent activity.

An important strength of the present study is the use of two separate population-based samples of adolescents from a county that is considered to be representative of Sweden as a whole because of its distribution of education, income, and employment levels together with urban and rural areas (SCB 2009). Because of the relatively representative population samples, our results might also be valid for other non-referred adolescent populations.

## Conclusions

To our knowledge, this is the first study to investigate the susceptibility properties of the 5HTTLPR in relation to emotion-regulated phenotypes of both depressive symptoms and delinquent behaviour in the same sample. Moreover, the findings are similarly presented in two separate adolescent community samples. If the assumption that the 5HTTLPR induces differential susceptibility to both positive and negative environmental influences is correct, the previous failures to measure and control for positive environmental factors might be a possible explanation for former inconsistent findings within the research field.
